# Hemoglobin-to-Red Cell Distribution Width Ratio Was Associated with Cardiovascular Diseases and Death

**DOI:** 10.3390/jcm14134464

**Published:** 2025-06-23

**Authors:** Mengyi Li, Hanbin Li, Wen Zhong, Shiqi Wang, Rui Liu, Hongxin Cheng, Lijuan Li, Quan Wei, Lu Wang

**Affiliations:** 1 Rehabilitation Medicine Center and Institute of Rehabilitation Medicine, West China Hospital, Sichuan University, Chengdu 610041, China; 18070224851@163.com (M.L.); hanbinli2002_119@163.com (H.L.); zhongwenzzww@163.com (W.Z.); wsqwangshiqi1@163.com (S.W.); liurui_96@163.com (R.L.); 13527420056@163.com (H.C.); lijuan-li@scu.edu.cn (L.L.); 2 Key Laboratory of Rehabilitation Medicine in Sichuan Province, Chengdu 610041, China

**Keywords:** hemoglobin-to-red blood cell distribution width ratio, cardiovascular diseases, death

## Abstract

**Background/Objectives:** The hemoglobin-to-red blood cell distribution width (RDW) ratio (HRR) reflects the status of inflammation and oxidative stress size. Previously, it has been suggested that HRR is associated with cardiovascular diseases (CVD). However, evidence has been limited for examining the association between HRR and the incidence of specific cardiovascular events (e.g., cardiovascular disease, stroke, congestive heart failure) and all-cause cardiovascular death and non-cardiovascular death, adjusting for known confounders. **Methods**: Data from the National Health and Nutrition Examination Survey (NHANES) in the year cycle of 1999–2018 were collected. HRR was calculated as the ratio of hemoglobin divided by the RDW. The outcomes were CVD, including stroke, congestive heart failure, atherosclerotic cardiovascular diseases (ASCVD), coronary artery disease as well as all-cause death including cardiovascular death and non-cardiovascular death. Univariate and multivariate analyses were performed to explore the association between HRR and outcomes. Restricted cubic spline curves were delineated. **Results**: In total, 47,719 participants were eligible for further analysis. In multivariate analysis adjusting for all confounding factors, higher HRR levels were significantly associated with a decreased risk of CVD. Compared to Q1 (<9.86), the odds ratio (OR) and 95% confidence intervals (95% CI) in Q2 (9.86–10.96), Q3 (10.96–11.97), and Q4 (≥11.97) were 0.79 (0.66, 0.94), 0.59 (0.48, 0.73), and 0.53, (0.42, 0.67), respectively, for predicting CVD. Similar results were observed for different subtypes of CVD, including stroke, congestive heart failure, and ASCVD. Notably, for predicting coronary heart disease, only Q3 was significant compared to Q1 (0.70, [0.54, 0.92], *p* = 0.010). HRR was significant for predicting all-cause death, cardiovascular death, and non-cardiovascular death. Additionally, HRR had the highest discriminative ability for predicting all-cause death compared with that of hemoglobin and RDW. **Conclusions**: A higher HRR was associated with a lower risk of CVD and death. Moderate levels of HRR were associated with the lowest risk for coronary heart disease. HRR had better discriminative ability than hemoglobin and RDW.

## 1. Introduction

As the most significant indicator in red blood cells (RBCs), hemoglobin serves as a dual indicator of reflecting the oxygen-carrying ability and the severity of anemia. The relationship between the level of hemoglobin and cardiovascular diseases (CVD) and death in the general population is inconclusive. Anemia demonstrates a high prevalence in patients with CVD and independently predicts adverse clinical outcomes [1]. For example, the prevalence of anemia is nearly 43% in patients with acute coronary syndrome, 70% in patients with heart failure [2,3], and 15–29% in patients with acute stroke [4]. Anemia causes ventricular remodeling and cardiac dysfunction [5], promotes the development of hyperemia-related emboli, increases the release of inflammatory mediators, the alteration of blood viscosity, and increases tissue hypoxia [6], thereby potentially increasing the risk of CVD or death. Therefore, most studies report that low hemoglobin can potentially be used to predict a poor prognosis of CVD [7]. Baseline low hemoglobin serves as a significant predictor of poor prognosis, including mortality and major adverse cardiovascular events in patients with percutaneous coronary intervention [8,9].

Another important indicator for RBCs is red blood cell distribution width (RDW). RDW is a routine component in blood tests, specifically measuring the variation in size among peripheral RBCs. The greater the disparity in RBC sizes within the bloodstream, the higher the RDW value. RDW is also used to diagnosis anemia, based on its role of reflecting the degree of anisocytosis [10]. Anisocytosis, a marker of disrupted erythrocyte homeostasis caused by impaired RBC production and survival, is clinically quantified as elevated RDW. The increased RDW levels are due to many biological processes including anemia, hematopoiesis abnormalities, inflammation, oxidative stress, chronic disease, nutritional deficiencies, and aging [10]. RDW serves as a nonspecific biomarker associated with multiple pathophysiological states and a prognostic indicator for adverse clinical outcomes. Additionally, a growing body of research reports that RDW is a novel marker for CVD, considering that CVDs are inflammation-related diseases. As RDW levels increase, the risk of CVD and death gradually increase [11,12,13,14,15,16,17,18].

However, the predictive value of one single parameter is limited and low. Hb levels alone are susceptible to the type of anemia, nutritional status, and therapeutic interventions and do not independently reflect the systemic oxidative stress load associated with inflammation [19]. Elevated RDW has been studied in many diseases and is not specific enough to be judged [20]. In comparison, the hemoglobin-to-RDW ratio (HRR), combining the predictive information from hemoglobin and RDW, integrates long-term adaptations to inflammation (e.g., anemia) and short-term stress (e.g., erythrocyte heterogeneity). It has been found that HRR is associated with the levels of inflammatory markers [21] (e.g., high-sensitivity C-reactive protein, erythrocyte sedimentation rate, interleukin-6, tumor necrosis factor-alpha). Therefore, it may more accurately represent the degree of systemic inflammatory response and the body’s oxidative stress [22]. Additionally, HRR is more informative in identifying patients with a high risk of CVD [23]. Studies have suggested that HRR can serve as a novel predictive biomarker for CVD, including stroke, coronary heart disease, heart failure [24,25,26,27,28], and other different types of vascular diseases [24,29,30]. Specifically, in a study of patients with heart failure, HRR was strongly associated with the prognosis of heart failure and was an important indicator for assessing the survival of patients [31]. Furthermore, HRR was negatively correlated with arterial stiffness, suggesting that it may predict cardiovascular disease risk by influencing the function of the autonomic nervous system [24]. However, most previous studies focused on the role of HRR in patients with these CVDs, and there is no evidence in the general population. Additionally, previous studies did not present whether HRR had better predictive value than hemoglobin and RDW. Thus, we collected data from the National Health and Nutrition Examination Survey (NHANES) study to explore the association between HRR values, CVD, and all-cause death and to compare the predictive values among the parameters.

## 2. Materials and Methods

### 2.1. NHANES Study

The NHANES study reported information on the representative sample of the US population biennially since 1999. Dietary habits, nutritional status, medical conditions, physical examination, and laboratory tests were collected, and the corresponding data are available at its website (https://wwwn.cdc.gov/nchs/nhanes/Default.aspx [accessed on 22 May 2024]). The study design and laboratory methods’ protocol have been published previously. This research was conducted in accordance with the Declaration of Helsinki and national standards. This survey was approved by National Center for Health Statistics Research Ethics Review Board. Informed consent was obtained from individual or guardian participants. NHANES was approved by the US National Center for Health Statistics Research Ethics Review Board (Protocol No. 98-12, Protocol No. 2011-17, Continuation of Protocol No. 2011-17, Protocol No. 2018-01) (available at: https://www.cdc.gov/nchs/nhanes/about/erb.html [accessed on 22 May 2024]).

### 2.2. Data Collection

We selected participants older than 18 years old in the year cycles of 1999–2000, 2001–2002, 2003–2004, 2005–2006, 2007–2008, 2009–2010, 2011–2012, 2013–2014, 2015–2016, and 2017–2018. The exclusion criteria included lack of the participants’ demographic data and other crucial factors. Multiple covariates were divided into four categories: demographic characteristics included age, sex, race (non-Hispanic white, non-Hispanic black, other Hispanic, Mexican American, and other races), socioeconomic status included education (below high school and high school or above) and marital status (married/living with partner, widowed/divorced/separated, and never married). The lifestyle factors included smoking status (never, mild, moderate, heavy, former) and drinking status (never, former, now), which were collected through self-reported questionnaires [32]. Former drinker was defined as having more than 1 drink per week in their lifetime and not drinking in the last year [33]. Health status indicators included some chronic diseases. The participants were asked if they have been told by physicians that they have diabetes, hypertension, hyperlipidemia, and chronic kidney disease (CKD). In addition, some physiological indicators, including white blood cells, platelet count, glucose, high-density lipoprotein, low-density lipoprotein, total cholesterol, and triglyceride were measured in the NHANES laboratory. The NHANES mortality data were collected from the 1986–2018 NHIS, the 1999–2018 NHANES, and public use link death files for NHANES III [34].

### 2.3. HRR Measurement

The NHANES had a detailed protocol for blood sample collection and detection [35]. The complete blood count tests were conducted using an automated Coulter Model S-Plus Jr (Coulter Electronics, Hialeah, FL, USA) [18]. HRR was measured as the ratio of hemoglobin divided by RDW (hemoglobin [g/L]/RDW [%]). Other laboratory tests such as glucose and lipid profiles were detected by an automatic analyzer (Boehringer Mannheim Diagnostics, Indianapolis, IN) [18].

### 2.4. Statistical Analysis

The complex survey design was used through primary sampling units, sample weights, and strata. All analyses were performed after sample weighting. We categorized patients with and without CVD. Weighted mean and weighted percentages with 95% confidence intervals (CIs) were used to describe the characteristics between a continuous variable and categorical variable, respectively. All data were normally distributed after weighting. T-test and χ^2^ test were used for comparing the differences between variables.

CVD was diagnosed through a two-step process combining self-reported medical history and structured questionnaire data. The participants were asked a question about five specific cardiovascular conditions: “Has a doctor or other healthcare professional ever diagnosed you with congestive heart failure, coronary heart disease, angina pectoris, a heart attack or a stroke?” If a participant answered “yes” to any of the cardiovascular conditions, he or she was diagnosed as having a CVD [36]. We performed logistic regression for the association between HRR and CVD and subtypes. To estimate the correlation between HRR and the risk of CVD, odds ratios (ORs) and 95% CIs were reported in weighted univariable and multivariable logistic analyses using a complex survey design. Cox regression analysis was conducted to calculate the HR and 95% CI of HRR and death, cardiovascular death, as well as non-cardiovascular death. HRR was treated as a continuous variable firstly. Then, it was classified into quartiles (Q1 = 1.99–9.86, Q2 = 9.86–10.96, Q3 = 10.96–11.97, Q4 = 11.97–16.42) and included as a categorical variable in weighted regression analysis. The lowest quartile (Q1) was set as the reference group to estimate the hazard ratios (HRs) and 95% CIs between the 2nd quartile (Q2), 3rd quartile (Q3), and 4th quartile (Q4) of HRR for predicting different outcomes.

For the multivariable regression analyses, we included variables with *p*-value < 0.05 in the univariate analysis. In model 1, we adjusted for age and sex; in model 2, we adjusted for factors in model 1 plus other correlated factors including race, education, marital status, drinking, smoking, diabetes, hypertension, hyperlipidemia, CKD, white blood cells, platelet count, glucose, high-density lipoprotein, low-density lipoprotein, total cholesterol, and triglyceride. In the multivariate regression analysis, some covariates had missing values with <5% missingness for all variables. To minimize potential bias, we employed multiple imputation with chained equations to handle missing data for the adjusted confounders and reduce bias. In addition, *p* for the trend was calculated by including the median HRR value in each group as a continuous variable in the models. Restricted cubic splines at 4 knots were illustrated to present the pattern and magnitude of the association between the HRR and each outcome. Kaplan–Meier curves were delineated to illustrate the cumulative survival probabilities among the quartile of HRR levels. Lastly, net reclassification improvement and integrated discrimination improvement indexes were calculated to compare the prognostic values between hemoglobin, RDW, and HRR. The statistical analyses were conducted using R version 4.4.2. A two side *p* < 0.05 was considered statistically significant.

## 3. Results

There were 94,737 participants in the year cycle of NHANES 1999–2020. We subsequently excluded 7535 participants lacking HRR measurements, 33,844 without mortality data, 3678 with missing CVD data, and 1961 aged below 18 years. After excluding participants without collected information, 47,719 participants were included in the final analysis. After weighting, the study represented 180,538,467 participants. Among them, 8.63% (8.24–9.04%) developed CVD, with 2.82% (2.64–3.02%) having stroke, 2.32% (2.16–2.50%) having congestive heart failure, 7.89% (7.52–8.29%) having atherosclerotic cardiovascular diseases (ASCVD), and 3.55% (3.30–3.83) having coronary heart disease. Among those included in the study, 11.31% (10.75–11.91%) died, 3.44% (3.17–3.73%) due to CVD and 7.87% (7.48–8.28%) due to non-CVD.

Table 1 shows the baseline characteristics of participants with and without CVD. In addition, Supplementary Tables S1 and S2 present the baseline characteristics of participants by HRR quartiles and mortality status, respectively. We found that participants with CVD were older, more likely to be female, more likely to be non-Hispanic White, and less likely to be other Hispanic, Mexican American, and other races. Participants with CVD were more likely to have an education status of below high school, less likely to be married/living with a partner, and more likely to be former drinkers and smokers. Participants with CVD had a higher probability of diabetes, hypertension, hyperlipidemia, and CKD (*p* < 0.001, Table 1). Additionally, participants with CVD tended to have higher levels of white blood cells, glucose, triglycerides, and RDW, as well as lower levels of platelet count, high-density lipoprotein, low-density lipoprotein, total cholesterol, and hemoglobin than participants without CVD.

In univariable analysis, higher HRR levels predicted lower risks of CVD. Further adjusting for age and sex in model 1 slightly alleviated the protective effect. In model 2, HRR per one-unit increment decreased the risk of CVD by 15% (0.85 [0.81, 0.89; <0.001]), the risk of stroke by 15% (0.85 [0.81, 0.90; <0.001]), the risk of congestive heart failure by 27% (0.76 [0.69, 0.78; <0.001]), the risk of ASCVD by 12% (0.88 [0.84, 0.92; <0.001]), and the risk of coronary heart disease by 9% (0.91 [0.86, 0.98; 0.009]) after further adjusting for all potential confounding factors (model 2, Table 2).

**Figure 1:**
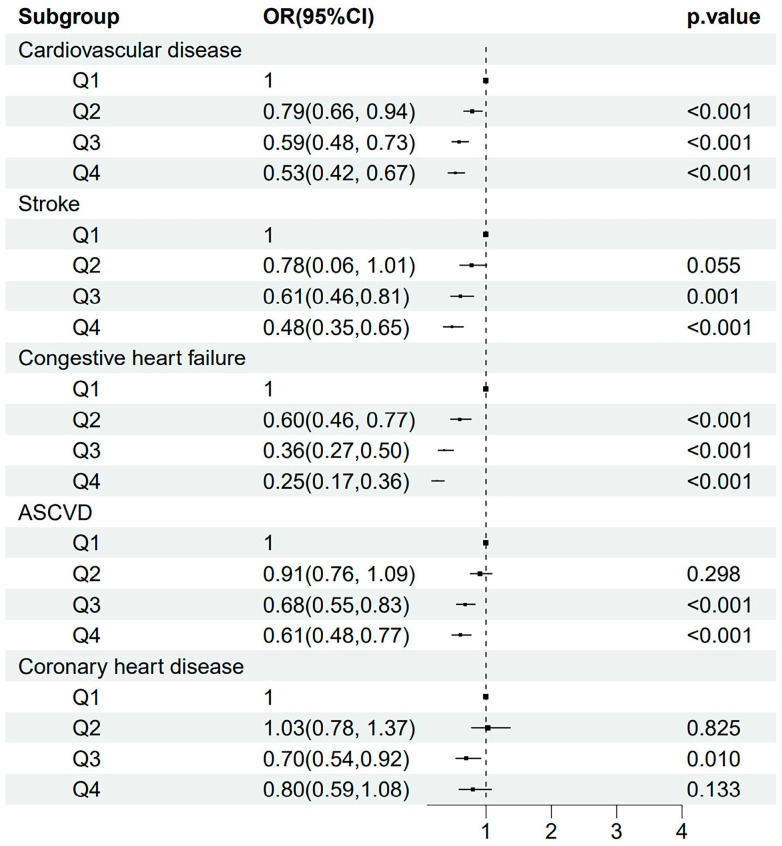
The odds ratios and 95% confidence intervals for quartiles of HRR predicting the risk of CVD, stroke, congestive heart failure, ASCVD, and coronary heart disease. Abbreviations: HRR: hemoglobin-to-red cell distribution width ratio; CVD: cardiovascular disease; ASCVD: atherosclerotic cardiovascular disease.

**Figure 2:**
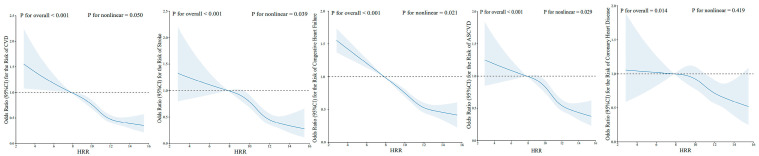
Restricted cubic spline curve for the relationship between HRR and the risk of cardiovascular diseases, stroke, congestive heart failure, ASCVD, and coronary heart disease. Abbreviations: HRR: hemoglobin-to-red cell distribution width ratio; ASCVD: atherosclerotic cardiovascular disease.

Then, we explored whether HRR quartiles were also associated with the risk of CVD and its different subtypes. The risk of CVD decreased along with the increment of HRR quartiles (all *p* for trend <0.001). A decreased OR (95% CI; *p*-value) was observed for HRR quartiles predicting CVD (*p* for trend <0.001): Q2 versus Q1 (0.79 [0.66, 0.94; 0.009]), Q3 versus Q1 (0.59 [0.48, 0.73; <0.001]), and Q4 versus Q1 (0.53 [0.42, 0.67; <0.001]). Similarly, a gradually decreased OR was also observed across HRR quartiles for predicting stroke, congestive heart failure, and ASCVD (Figure 1). Correspondingly, Figure 2 demonstrates a similar trend of a J-shaped dose-dependent relationship between HRR levels and CVD, stroke, congestive heart failure, as well as ASCVD. However, for predicting coronary heart disease, the ORs for Q2 versus Q1 and Q4 versus Q1 were insignificant, except for the OR of Q3 versus Q1 (0.70 [0.54, 0.92]; 0.010). Figure 2 suggested that the Q3 of HRR (10.96–11.97) had the lowest risk of coronary heart disease, and an increased risk of coronary heart disease was observed when HRR gradually increased after Q3.

Moreover, in univariate analysis and multivariate analysis after adjusting for age and sex (model 1), a one-unit HRR increase was associated with a lower risk of all-cause death. In model 2 after adjusting for all potential confounding factors, one-unit increment of HRR was associated with a risk decrease of all-cause death of 18% (HR 0.82, 95% CI [0.79, 0.85], Table 3). However, the association was also significant for one-unit of HRR change and cardiovascular death as well as non-cardiovascular death in model 2 (*p* < 0.001, Table 3).

**Figure 3:**
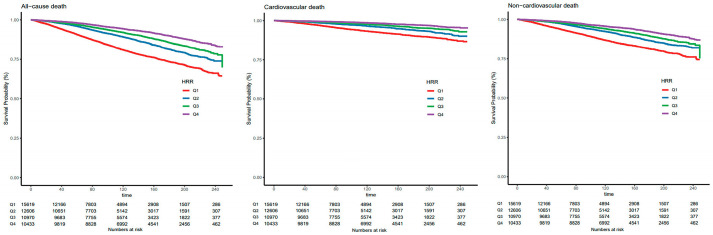
The Kaplan–Meier curves show the cumulative survival probabilities for all-cause death, cardiovascular death, and non-cardiovascular death in quartiles of HRR levels. Abbreviations: Q1: quartile 1; Q2: quartile 2; Q3: quartile 3; Q4: quartile 4; HRR, hemoglobin-to-red cell distribution width ratio.

**Figure 4:**
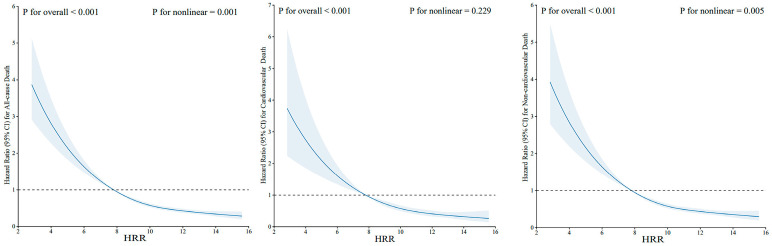
Restricted cubic spline curve for the relationship between HRR and risk of all-cause death, cardiovascular death, and non-cardiovascular death. Abbreviations: HRR: hemoglobin-to-red cell distribution width ratio.

When HRR was regarded as a categorical variable, in univariate analysis, HRs and 95% CIs were decreased along with the increase of HRR quartiles. Furthermore, in model 2 further adjusting for all factors, similar results were shown. The adjusted HRs were significant for the quartiles of HRR for predicting all-cause death (Q2 versus Q1: 0.66 [0.58, 0.74], Q3 versus Q1: 0.62 [0.53, 0.74], Q4 versus Q1: 0.52 [0.44, 0.61]). For cardiovascular death and non-cardiovascular death, similar results were observed. Kaplan–Meier curves were created to illustrate survival based on HRR levels and demonstrated a progressive increase in survival probability across quartiles (Q1, Q2, Q3, Q4) of HRR, with higher quartiles associated with improved survival outcomes. Q4 of HRR had the lowest mortality and Q1 had the highest mortality in Figure 3, with the *p*-value for the log-rank test being <0.001 for all-cause death, cardiovascular death, and non-cardiovascular death. Restricted cubic spline curves in Figure 4 present the dose–response relationship for all-cause death, cardiovascular death, and non-cardiovascular death along with the increment of HRR levels. Additionally, the model with HRR added into the basic model had the highest net reclassification improvement index (0.095, 95% CI 0.069, 0.121) and integrated discrimination improvement index (0.009, 95% CI 0.007, 0.011, Table 4).

Additionally, we performed receiver operating characteristic (ROC) analysis, and the ROC curves for CVD are shown in Supplementary Figure S1. The area under the curve (AUC) for the ROC curve of HRR was 0.611 (95% CI: 0.603–0.619) for predicting CVD and 0.675 (95% CI: 0.662–0.688) for congestive heart failure. Similarly, the ROC curve of HRR for predicting death is presented in Supplementary Figure S2. ROC analysis revealed that the predictive performance of HRR, as measured by the AUC, was 0.564 (95% CI: 0.557–0.571) for overall death, higher at 0.574 (95% CI: 0.563–0.586) for cardiac-related deaths, and the lowest at 0.552 (95% CI: 0.544–0.560) for non-cardiac deaths. These results demonstrate that HRR showed slightly stronger predictive value for CVD than for death, with the highest predictive performance observed for congestive heart failure.

To further investigate the key factors influencing hemoglobin, RDW, and HRR, we presented the values of hemoglobin, RDW, and HRR across different states of inflammation, anemia status, and comorbidities, including diabetes, hypertension, hyperlipidemia, and CKD in Supplementary Table S3 and conducted sensitivity and subgroup analyses (Supplementary Tables S4–S6). Supplementary Tables S4 and S5 display the sensitivity analysis after adding CRP or anemia in multivariate analysis of HRR predicting various CVDs and deaths. For CVDs, the ORs were 0.85 (95% CI: 0.82–0.88) when CRP was added and 0.83 (95% CI: 0.79–0.87) when anemia was included. Similar trends were observed for stroke, congestive heart failure, ASCVD, and coronary heart disease. HRR had consistent protective effects against all-cause death, cardiovascular death, and non-cardiovascular death, as evidenced by HRs below 1 and significant *p*-values (*p* < 0.001). The results demonstrated that while inflammation, anemia, and comorbidities may affect hemoglobin, RDW, and HRR levels, they did not significantly interact with the independent associations between HRR and CVDs or between HRR and death. Subgroup analysis grouped by various variables is exhibited in Supplementary Table S6. In most subgroups, higher HRR was significantly associated with a lower risk of CVD and a lower risk of all-cause death. Additionally, we found that there was a significant interaction of sex with HRR in predicting CVD (*p* for interaction = 0.04) and all-cause death (*p* for interaction < 0.01).

## 4. Discussion

Our study clearly showed that higher HRR levels were associated with a lower prevalence of CVD, stroke, congestive heart failure, and ASCVD. However, this relationship was not fully consistent regarding coronary heart disease. Specifically, the fourth quartile of HRR in all subgroup analysis suggested a significantly higher CVD, stroke, congestive heart failure, ASCVD prevalence compared with that in the first quartile, while HRR levels in the moderate range predicted the lowest risk of coronary heart disease. In addition, after adjusting for multiple covariates, including demographic variables, lifestyle factors, and blood index counts, this association still remained. We clearly revealed that a lower HRR was positively associated with an increased risk of all causes of death. After adding parameters into the basic model, HRR’s discriminative ability was better than that of hemoglobin and RDW. Our study’s results are in line with those of previous studies, which reported that lower quantiles of HRR were related to poor prognosis [31,37]. For example, Xiu et al. identified that a lower HRR (<10.25) predicts higher all-cause and cardiac mortality in patients following a percutaneous coronary intervention [24]. In addition, a study [38] has indicated that low HRR levels are associated with higher severity and mortality in stroke patients and showed that HRR outperformed Hb and RDW alone in predicting mortality (AUC: 0.975, 0.952, and 0.911, respectively).

Interestingly, our results showed that compared with the lowest quartile, the highest quartile of HRR was not significantly associated with a decreased risk of coronary heart disease. However, a previous study [39] has indicated that a one-unit increase in HRR is associated with a 49% reduced likelihood of coronary heart disease, and beyond an HRR of 1.02, this reduction jumps to 91%. We found that this article was inconsistent with the criteria for Q1, Q2, Q3, and Q4 as defined in our article, which may explain the different results. In addition, the populations included in the two studies are not exactly the same, differing in sociodemographic and health-related characteristics. The mechanism of the role of HRR on CVD is still not clear, as different studies have indicated that the role played by HRR varies considerably in different disease contexts. For instance, a study [39] has shown that HRR is related to poorer outcomes of nasopharyngeal carcinoma. Furthermore, patients of different age, gender, and ethnic backgrounds may have different responses to HRR [40]. Ying et al. found that HRR showed a stronger age-dependent association with in-hospital mortality in chronic heart failure patients [41]. Among individuals with heart failure, those with a reduced ejection fraction showed higher all-cause mortality than those with middle-range ejection and a preserved ejection fraction did, but only in males [42]. In summary, the relationship between HRR and the risk and prognosis of CVD, especially coronary heart disease, requires further investigation.

The specific mechanism of HRR on CVD may be explained by several possible reasons. Firstly, HRR may reflect the degree of hypoxia in tissues. The main function of RBC is delivering oxygen. A low HRR may indicate impaired oxygen carrying capacity, vascular dysregulation, and changes in viscosity or blood flow patterns [43]. In patients with a low HRR, when an artery has stenosis, the amount of oxygen supplied in the downstream is significantly reduced. To compensate, the heart rate would increase to get adequate oxygen delivery. The high oxygen demand and severe heart burden may lead to ventricular remodeling and hypertrophy, aggravating the occurrence of cardiovascular events [44]. Moreover, a higher level of RDW is related to higher total cholesterol erythrocyte membrane levels [45], which result in decreased RBC deformability [46] and reduce the fluidity of the RBC membrane [46], leading to a lower lifespan of circulating RBCs, ineffective RBC renewal, and impaired blood flow in microcirculation [47,48]. In addition, in laboratory tests, RDW is a key parameter for measuring the heterogeneity of RBC sizes and shapes. The larger the value of RDW, the greater the variability in the size and shape of the RBCs in the blood sample. The normal range is usually between 11% and 15% [49]. When RDW exceeds normal values, it may indicate the presence of various types of anemia, hematopoietic abnormalities, or congenital RBC abnormalities. In contrast, a smaller RDW indicates a more consistent size and shape of the RBCs [24]. Thus, lower HRR levels hint at poorer RBC deformability and higher risks of anemia, leading to a higher risk of CVD.

Another reason has been attributed to the underlying inflammation [50]. Anemia of inflammation is another cause of anemia, which is mediated by inflammatory cytokines [51]. Hemoglobin and RDW have been reported to be associated with inflammatory cytokines, including tumor necrosis factor-α, and interleukin [15,52,53]. These inflammatory cytokines are associated with a poor prognosis [54]. Furthermore, in a systemic inflammatory response, neutrophils are usually increased while lymphocytes are decreased, resulting in an elevated neutrophil-to-lymphocyte ratio (NLR), thus reflecting the severity of systematic inflammation [55]. The NLR also predicts the severity and prognosis of cardiovascular disease, and studies have shown that the NLR is strongly associated with a poor prognosis in coronary heart disease [56], heart failure [57], peripheral artery disease [58], and acute myocardial infarction [59]. Future studies are needed to further explore whether combining the NLR and HRR (or other biomarkers) can enhance the predictive ability for CVDs and death.

Systemic inflammation may activate macrophages and increase the expression of adhesion molecules on the erythrocyte membrane, resulting in increased adherence of RBCs to the endothelium [60], which may also promote the clearance of RBCs and the decrease of their lifespan [61]. Additionally, inflammation may inhibit iron utilization, leading to ineffective erythropoiesis [60]. Ineffective erythropoiesis reduces the generation of RBCs with normal function and the response of bone marrow to erythropoietin, leading to impaired hematopoietic function [46,62]. Therefore, inflammation and oxidative stress promote the clearance of RBC and prevent the utilization of erythropoietin and iron, and are associated with a lower level of hemoglobin and a higher level of RDW. A decreased HRR reflects decreased hemoglobin and increased RDW due to an elevated degree of inflammation and oxidative stress, which was associated with higher risks of CVD, stroke, congestive heart failure, and ASCVD.

Specifically, decreased hemoglobin and increased RDW is related to decreased “antioxidant” function of RBCs because the loss of RBC deformability and changes in erythrocyte homogeneity would destroy the transport of RBCs in blood vessels [63], thus creating an increased oxidative burden that accelerates the development of atherosclerosis [64,65]. In summary, a lower HRR reflect higher degrees of hypoxia, lipid disorder, inflammation, oxidative stress, and thrombosis. These pathophysiological processes can hinder erythropoiesis and impede the maturation of erythrocytes, resulting in anisocytosis and decreased oxygen supply, and promoting the development of CVD. In our study, we found a negative correlation between HRR and CRP, with a correlation coefficient of –0.178, which is inconsistent with a previous study [21] finding that the higher the HRR, the higher the levels of inflammatory markers. There are several reasons that may explain this inconsistency. Firstly, the populations enrolled in diverse studies exhibited heterogeneous underlying disease backgrounds. Secondly, the confounders corrected for in the study design were different.

In addition, regarding the interaction between sex and HRR, there may be the following reasons: firstly, sex hormones play an important role in cardiovascular disease. Estrogens have a cardiovascular protective effect and reduce the risk of atherosclerosis, whereas androgens may increase the risk of CVD [66]. Secondly, women typically have a weaker inflammatory response and higher antioxidant capacity compared to those in men, which may reduce damage to the cardiovascular system from oxidative stress [67].

The results of this study have some clinical significance. HRR suggests the average volume and heterogeneity of RBC and provides an effective and stable assessment by combining hemoglobin and RDW, potentially serving as a better predictor. A low HRR reflects decreased oxygen carrying function, poor erythropoiesis function of bone marrow, poor physical status, and higher risk of clinical deterioration. In this study, a low HRR acted as a predictive tool to identify patients with an elevated risk of CVD and death. Our results gave a hint about the characteristics of HRR in patients with CVD. Additionally, HRR measurements are relatively simple and cost-effective, making them highly accessible and potentially valuable in resource-limited settings. Therefore, HRR can provide supplementary information alongside traditional detection metrics, thereby improving the early detection and comprehensive assessment for stratification of patients with high risk of CVD and death [51].

However, this study also has several limitations: Firstly, this study design precludes definitive causal inferences between HRR and the risk and prognosis of CVD due to the observational nature of the NHANES study. Further studies are needed to explore the causal relationship between HRR and CVD. Secondly, a single measurement of hemoglobin and RDW may not be representative of long-term changes, and some of the data relied on participant self-reports, such as CVD, smoking and alcohol consumption, which may have inaccurate recalls. Thirdly, while we controlled for multiple covariates, there may be other confounding factors (e.g., physical activity level, drug consumption) that could influence the relationship between HRR and CVD, which were not accounted for in this study. Finally, although the NHANES dataset has a large sample size, when performing detailed subgroup analyses, the sample size may be relatively small for some specific subgroups, resulting in less than effective analyses.

## 5. Conclusions

Our study found that a higher HRR level was associated with a decreased risk of CVD and death, including all-cause death, cardiovascular death, and non-cardiovascular death in the general population. Therefore, to some extent, HRR can be used as a simple and inexpensive biomarker for CVD risk assessment. Moderate levels of HRR were associated with the lowest risk of coronary heart disease. Further studies are needed to provide more evidence about the role of HRR levels in the development and prognosis of different CVDs, especially coronary heart disease.

## Figures and Tables

**Table 1:** Patient characteristics, stratified by with and without CVD.

Variables	Overall (47,719) N = 207,540,003	Without CVD (42,474) N = 189,102,339	With CVD (5245) N = 18,437,664	*p*-Value
Age, years	47.2 (17.0)	45.4 (16.2)	64.8 (13.6)	<0.001
Female	25,780 (52%)	23,331 (53%)	2449 (47%)	<0.001
Race				<0.001
Non-Hispanic White	22,089 (69%)	18,949 (68%)	3140 (76%)	
Non-Hispanic Black	10,131 (11%)	8979 (11%)	1152 (11%)	
Other Hispanic	4094 (5.6%)	3751 (5.8%)	343 (3.4%)	
Mexican American	8776 (8.2%)	8125 (8.6%)	651 (4.2%)	
Other Races	4539 (6.9%)	4215 (7.0%)	324 (5.9%)	
Education				<0.001
Below high school	13,438 (17%)	11,430 (16%)	2008 (26%)	
High school or above	36,121 (83%)	32,534 (84%)	3587 (74%)	
Marital status				<0.001
Married/Living with partner	29,718 (64%)	26,632 (64%)	3086 (60%)	
Widowed/Divorced/Separated	10,943 (19%)	8846 (17%)	2097 (34%)	
Never married	8488 (17%)	8111 (19%)	377 (6.5%)	
Drinking				<0.001
Never	6579 (12%)	5814 (11%)	765 (14%)	
Mild	14,692 (36%)	13,033 (36%)	1659 (37%)	
Moderate	6607 (17%)	6207 (18%)	400 (8.7%)	
Heavy	8832 (21%)	8327 (22%)	505 (11%)	
Former	7951 (15%)	6295 (13%)	1656 (30%)	
Smoking				<0.001
Never	26,940 (54%)	24,733 (55%)	2207 (39%)	
Former	12,315 (25%)	10,026 (23%)	2289 (40%)	
Now	10,331 (21%)	9220 (21%)	1111 (21%)	
Vascular diseases				
Diabetes	8665 (13%)	6425 (11%)	2240 (36%)	<0.001
Hypertension	21,295 (38%)	16,783 (34%)	4512 (77%)	<0.001
Hyperlipidemia	35,621 (71%)	30,794 (69%)	4827 (88%)	<0.001
Chronic kidney diseases	9133 (15%)	6572 (12%)	2561 (41%)	<0.001
Laboratory tests				
White blood cells, 1000 cells/uL	7.30 (2.50)	7.27 (2.44)	7.54 (3.05)	<0.001
Platelet count, 1000 cells/uL	254 (66)	255 (65)	237 (72)	<0.001
Glucose, mg/dL	105 (31)	104 (29)	120 (43)	<0.001
HDL, mg/dL	53 (16)	53 (16)	50 (16)	<0.001
LDL, mg/dL	116 (36)	117 (35)	104 (39)	<0.001
Triglyceride, mg/dL	133 (114)	131 (115)	150 (105)	<0.001
Total cholesterol, mg/dL	196 (42)	197 (41)	186 (46)	<0.001
Hemoglobin, g/L	143 (15)	143(15)	140 (16)	<0.001
RDW (%)	13.03 (1.24)	12.98 (1.21)	13.58 (1.46)	<0.001
HRR (g/L/%)	11.08 (1.63)	11.14 (1.61)	10.46 (1.71)	<0.001

Abbreviation: N: weighted number in each group; CVD, cardiovascular diseases; HDL, high-density lipoprotein; LDL, low-density lipoprotein; RDW, red cell distribution width; HRR, hemoglobin-to-red cell distribution width (RDW) ratio; the mean (SD) for continuous variables and weighted number (unweighted percentage) for categorical variables are shown.

**Table 2:** Odds ratios (95% CIs) of HRR as a continuous variable and categorical variable for predicting the risk of cardiovascular disease.

The Risk of Stroke	Univariate Analysis	Model 1	Model 2
Cardiovascular disease	0.79 (0.78, 0.81), <0.001	0.83 (0.80, 0.85), <0.001	0.85 (0.81, 0.89), <0.001
Cardiovascular disease			
Q1	Ref.	Ref.	Ref.
Q2	0.63 (0.57, 0.70), <0.001	0.68 (0.62, 0.75), <0.001	0.79 (0.66, 0.94), 0.009
Q3	0.46 (0.41, 0.52), <0.001	0.54 (0.48, 0.61), <0.001	0.59 (0.48, 0.73), <0.001
Q4	0.33 (0.30, 0.37), <0.001	0.46 (0.40, 0.52), <0.001	0.53 (0.42, 0.67), <0.001
*p* for trend	<0.001	<0.001	<0.001
			
Stroke	0.78 (0.76, 0.80), <0.001	0.84 (0.82, 0.87), <0.001	0.85 (0.81, 0.90), <0.001
Stroke			
Q1	Ref.	Ref.	Ref.
Q2	0.61 (0.52, 0.72), < 0.001	0.71 (0.60, 0.83), < 0.001	0.78 (0.60, 1.01), 0.055
Q3	0.40 (0.33, 0.48), <0.001	0.54 (0.45, 0.65), <0.001	0.61 (0.46, 0.81), 0.001
Q4	0.28 (0.23, 0.34), <0.001	0.49 (0.39, 0.61), <0.001	0.48 (0.35, 0.65), <0.001
*p* for trend	<0.001	<0.001	<0.001
			
Congestive heart failure	0.71 (0.69, 0.74), <0.001	0.73 (0.70, 0.75), <0.001	0.73 (0.69, 0.78), <0.001
Congestive heart failure			
Q1	Ref.	Ref.	Ref.
Q2	0.46 (0.39, 0.54), < 0.001	0.49 (0.42, 0.59), < 0.001	0.60 (0.46, 0.77), <0.001
Q3	0.25 (0.21, 0.30), <0.001	0.29 (0.24, 0.34), <0.001	0.36 (0.27, 0.50), <0.001
Q4	0.18 (0.15, 0.23), <0.001	0.24 (0.19, 0.30), <0.001	0.25 (0.17, 0.36), <0.001
*p* for trend	<0.001	<0.001	<0.001
			
ASCVD	0.81 (0.80, 0.83), <0.001	0.85 (0.83, 0.88), <0.001	0.88 (0.84, 0.92), <0.001
ASCVD			
Q1	Ref.	Ref.	Ref.
Q2	0.70 (0.63, 0.77), <0.001	0.77 (0.69, 0.85), <0.001	0.91 (0.76, 1.09), 0.298
Q3	0.52 (0.46, 0.58), <0.001	0.62 (0.54, 0.70), <0.001	0.68 (0.55, 0.83), <0.001
Q4	0.37 (0.33, 0.41), <0.001	0.51 (0.45, 0.58), <0.001	0.61 (0.48, 0.77), <0.001
*p* for trend	<0.001	<0.001	<0.001
			
Coronary heart disease	0.82 (0.79, 0.85), <0.001	0.85 (0.82, 0.89), <0.001	0.91 (0.86, 0.98), 0.009
Coronary heart disease			
Q1	Ref.	Ref.	Ref.
Q2	0.71 (0.61, 0.82), < 0.001	0.79 (0.68, 0.92), 0.003	1.03 (0.78, 1.37), 0.825
Q3	0.55 (0.46, 0.65), <0.001	0.64 (0.53, 0.78), <0.001	0.70 (0.54, 0.92), 0.010
Q4	0.39 (0.33, 0.46), <0.001	0.51 (0.42, 0.62), <0.001	0.80 (0.59, 1.08), 0.133
*p* for trend	<0.001	<0.001	0.021

Model 1 adjusted for age and sex; model 2 adjusted for model 1 plus race, education, marital status, drinking, smoking, diabetes, hypertension, hyperlipidemia, chronic kidney diseases, white blood cells, platelet count, glucose, HDL, LDL, total cholesterol, and triglyceride. Abbreviations: 95% CI, 95% confidence interval; HDL, high-density lipoprotein; LDL, low-density lipoprotein; Q1: quartile 1; Q2: quartile 2; Q3: quartile 3; Q4: quartile 4; HRR, hemoglobin-to-red cell distribution width ratio. ASCVD: atherosclerotic cardiovascular disease.

**Table 3:** Hazard ratios (95% CI) of HRR as a continuous variable and categorical variable for predicting the risk of death.

The Risk of Death	Univariate Analysis	Model 1	Model 2
All-cause death	0.78 (0.77, 0.80), <0.001	0.81 (0.79, 0.82), <0.001	0.82 (0.79, 0.85), <0.001
All-cause death			
Q1	Ref.	Ref.	Ref.
Q2	0.58 (0.54, 0.62), <0.001	0.61 (0.57, 0.66), <0.001	0.66 (0.58, 0.74), <0.001
Q3	0.45 (0.41, 0.49), <0.001	0.54 (0.49, 0.58), <0.001	0.62 (0.53, 0.74), <0.001
Q4	0.32 (0.29, 0.34), <0.001	0.47 (0.43, 0.51), <0.001	0.52 (0.44, 0.61), <0.001
*p* for trend	<0.001	<0.001	<0.001
			
Cardiovascular death	0.76 (0.74, 0.77), <0.001	0.79 (0.76, 0.81), <0.001	0.80 (0.75, 0.84), <0.001
Cardiovascular death			
Q1	Ref.	Ref.	Ref.
Q2	0.56 (0.49, 0.64), <0.001	0.59 (0.52, 0.67), <0.001	0.62 (0.51, 0.76), <0.001
Q3	0.38 (0.33, 0.44), <0.001	0.48 (0.42, 0.55), <0.001	0.56 (0.43, 0.73), <0.001
Q4	0.25 (0.21, 0.29), <0.001	0.40 (0.34, 0.46), <0.001	0.45 (0.33, 0.61), <0.001
*p* for trend	<0.001	<0.001	<0.001
			
Non-cardiovascular death	0.79 (0.78, 0.81), <0.001	0.82 (0.80, 0.84), <0.001	0.83 (0.79, 0.86), <0.001
Non-cardiovascular death			
Q1	Ref.	Ref.	Ref.
Q2	0.59 (0.55, 0.64), <0.001	0.62 (0.57, 0.68), <0.001	0.67 (0.59, 0.77), <0.001
Q3	0.48 (0.43, 0.53), <0.001	0.56 (0.51, 0.63), <0.001	0.65 (0.53, 0.80), <0.001
Q4	0.35 (0.32, 0.39), <0.001	0.51 (0.46, 0.56), <0.001	0.56 (0.46, 0.67), <0.001
*p* for trend	<0.001	<0.001	<0.001

Model 1 adjusted for age and sex; model 2 adjusted for age, sex, race, education, marital status, drinking, smoking, diabetes, hypertension, hyperlipidemia, chronic kidney diseases, white blood cells, platelet count, glucose, HDL, LDL, total cholesterol, and triglyceride. Abbreviations: 95% CI, 95% confidence interval; HDL, high-density lipoprotein; LDL, low-density lipoprotein; Q1: quartile 1; Q2: quartile 2; Q3: quartile 3; Q4: quartile 4; HRR, hemoglobin-to-red cell distribution width ratio.

**Table 4:** The prognostic value of hemoglobin, RDW, and HRR for all-cause death.

Variables	NRI	*p*-Value	IDI	*p*-Value
Basic model + hemoglobin	0.081 (0.055, 0.107)	<0.001	0.005 (0.003, 0.006)	<0.001
Basic model + RDW	0.092 (0.067, 0.117)	<0.001	0.007 (0.006, 0.009)	<0.001
Basic model + HRR	0.095 (0.069, 0.121)	<0.001	0.009 (0.007, 0.011)	<0.001

Abbreviations: NRI: net reclassification improvement, IDI: integrated discrimination improvement; RDW, red cell distribution width; HRR, hemoglobin-to-red cell distribution width ratio. The basic model included age, sex, race, education, marital status, drinking, smoking, diabetes, hypertension, hyperlipidemia, chronic kidney diseases, white blood cells, platelet count, glucose, HDL, LDL, total cholesterol, and triglyceride.

## Data Availability

The datasets generated in this study are available in the National Health and Nutrition Examination Survey (NHANES) repository, (https://www.cdc.gov/nchs/nhanes/ [accessed on 22 May 2024]).

## Supplementary Materials

The following supporting information can be downloaded at: https://www.mdpi.com/article/10.3390/jcm14134464/s1, Table S1: Patient characteristics, stratified by HRR quartiles; Table S2: Patient characteristics, stratified by all-cause death; Table S3: The value of hemoglobin, RDW, and HRR grouped by inflammation status, anemia status, and comorbidities including diabetes, hypertension, hyperlipidemia, and chronic kidney disease; Table S4: Sensitivity analysis after adding CRP or anemia in multivariate analysis of HRR prediction of various cardiovascular diseases; Table S5: Sensitivity analysis after adding CRP or anemia in multivariate analysis of HRR predicting various deaths; Table S6: Subgroup analysis grouped by age, sex, race, inflammation status, anemia status, and comorbidities including diabetes, hypertension, hyperlipidemia, and chronic kidney disease; Figure S1. The Receiver Operating Characteristic (ROC) curve of HRR (Hemoglobin-to-Red Cell Distribution Width ratio) for predicting cardiovascular diseases; Figure S2. The Receiver Operating Characteristic (ROC) of HRR (Hemoglobin-to-Red Cell Distribution Width ratio) for predicting death.

## Abbreviations

The following abbreviations are used in this manuscript:

RBC	red blood cell
RDW	red blood cell distribution width
HRR	hemoglobin-to-red cell distribution width ratio
NHANES	National Health and Nutrition Examination Survey
ASCVD	atherosclerotic cardiovascular disease
CVD	cardiovascular disease
CHD	coronary heart disease
CKD	chronic kidney disease
NLR	neutrophil-to-lymphocyte ratio
ROC	receiver operating characteristic
AUC	area under the curve
HRs	hazard ratios
CIs	confidence intervals
ORs	odds ratios
Q	quartile
