# Supplementary material for: Hemoglobin-to-Red Cell Distribution Width Ratio Was Associated with Cardiovascular Diseases and Death
Source: J Clin Med. 2025 Jun 23;14(13):4464. doi: 10.3390/jcm14134464 (PMC12250184; doi:10.3390/jcm14134464)
Supplement: Supplementary file 1 [file jcm-14-04464-s001.zip › jcm-3641134-supplementary.pdf]

Supplemental Materials

Supplementary Table S1: Patient characteristics, stratified by HRR quartiles.

Supplementary Table S2: Patient characteristics, stratified by all-cause death.

Supplementary Table S3: The value of hemoglobin, RDW, and HRR grouped by inflammation status, anemia status, and comorbidities including diabetes, hypertension, hyperlipidemia, and chronic kidney disease.

Supplementary Table S4: Sensitivity analysis after adding CRP or anemia in multivariate analysis of HRR prediction of various cardiovascular diseases.

Supplementary Table S5: Sensitivity analysis after adding CRP or anemia in multivariate analysis of HRR predicting various deaths.

Supplementary Table S6: Subgroup analysis grouped by age, sex, race, inflammation status, anemia status, and comorbidities including diabetes, hypertension, hyperlipidemia, and chronic kidney disease.

Supplementary Figure S1. The Receiver Operating Characteristic (ROC) curve of HRR (Hemoglobin-to-Red Cell Dis-tribution Width ratio) for predicting cardiovascular diseases.

Abbreviation: CVD, Cardiovascular Diseases; CHD, Congestive Heart Failure; ASCVD: Atherosclerotic Cardiovascular Diseases; CHD: Coronary Heart Disease.

Supplementary Figure S2. The Receiver Operating Characteristic (ROC) of HRR (Hemoglobin-to-Red Cell Distribution Width ratio) for predicting death.

Abbreviation: CVD death: Cardiovascular Death; Non-CVD death: Non-cardiovascular Death.

Supplementary Table S1. Patient characteristics, stratified by HRR quartiles.

Variables	Overall (47719)<br>N = 207,536,251	Q1 (15619)<br>N = 51,966,212	Q2 (12606)<br>N = 51,944,114	Q3 (10970)<br>N = 51,881,942	Q4 (10433)<br>N = 51,743,982	p-value
**Age, years**	47.2 (17.0)	51.3 (18.1)	48.8 (17.2)	46.3 (16.0)	42.3 (15.0)	<0.001
**Female**	25,779 (52%)	11,849 (80%)	7,830 (67%)	4,493 (45%)	1,607 (16%)	<0.001
**Race**						<0.001
Non-Hispanic White	22,089 (69%)	5,180 (56%)	5,646 (69%)	5,609 (74%)	5,654 (75%)	
Non-Hispanic Black	10,130 (11%)	5,267 (22%)	2,499 (11%)	1,480 (6.5%)	884 (4.1%)	
Other Hispanic	4,094 (5.6%)	1,374 (6.3%)	1,153 (5.8%)	882 (5.5%)	685 (4.9%)	
Mexican American	8,776 (8.2%)	2,294 (7.8%)	2,097 (7.6%)	1,993 (7.6%)	2,392 (9.7%)	
Other Races	4,539 (6.9%)	1,504 (7.9%)	1,211 (6.7%)	1,006 (6.5%)	818 (6.3%)	
**Education**						<0.001
Below high school	13,437 (17%)	4,553 (20%)	3,284 (16%)	2,828 (16%)	2,772 (17%)	
High school or above	36,121 (83%)	11,033 (80%)	9,303 (84%)	8,131 (84%)	7,654 (83%)	
**Marital status**						<0.001
Married/Living with partner	29,717 (64%)	8,411 (58%)	7,623 (65%)	6,986 (67%)	6,697 (65%)	
Widowed/Divorced/Separated	10,943 (19%)	4,609 (26%)	2,903 (20%)	2,034 (16%)	1,397 (13%)	
Never married	8,488 (17%)	2,472 (15%)	1,973 (16%)	1,828 (17%)	2,215 (22%)	
**Drinking**						<0.001
Never	6,579 (12%)	2,791 (17%)	1,797 (12%)	1,179 (9.6%)	812 (7.5%)	
Mild	14,692 (36%)	4,100 (33%)	3,724 (36%)	3,550 (38%)	3,318 (36%)	

**Variables**	**Overall (47719)** N = 207,536,251	**Q1 (15619)** N = 51,966,212	**Q2 (12606)** N = 51,944,114	**Q3 (10970)** N = 51,881,942	**Q4 (10433)** N = 51,743,982	**p-value**
Moderate	6,607 (17%)	1,955 (17%)	1,797 (19%)	1,540 (17%)	1,315 (15%)	
Heavy	8,832 (21%)	1,843 (15%)	1,972 (19%)	2,172 (22%)	2,845 (29%)	
Former	7,950 (15%)	2,930 (18%)	1,998 (14%)	1,590 (13%)	1,432 (13%)	
**Smoking**						<0.001
Never	26,940 (54%)	9,460 (60%)	7,091 (56%)	5,573 (51%)	4,816 (47%)	
Former	12,315 (25%)	3,779 (24%)	3,092 (25%)	2,854 (26%)	2,590 (24%)	
Now	10,330 (21%)	2,367 (15%)	2,413 (19%)	2,530 (23%)	3,020 (29%)	
**Vascular diseases**						
Diabetes	8,665 (13%)	3,749 (20%)	2,123 (13%)	1,532 (11%)	1,261 (9.2%)	<0.001
Hypertension	21,295 (38%)	8,040 (46%)	5,426 (38%)	4,238 (34%)	3,591 (33%)	<0.001
Hyperlipidemia	35,620 (71%)	11,316 (72%)	8,951 (70%)	7,799 (70%)	7,554 (72%)	0.028
Chronic kidney diseases	9,133 (15%)	4,218 (24%)	2,334 (16%)	1,434 (10%)	1,147 (8.9%)	<0.001
**Laboratory tests**						
White blood cells, 1000 cells/ul	7.30 (2.50)	7.36 (3.35)	7.25 (2.33)	7.24 (2.06)	7.34 (2.04)	0.003
Platelet count, 1000 cells/ul	254 (66)	262 (78)	253 (64)	251 (62)	248 (58)	<0.001
Glucose, mg/dL	105 (31)	106 (32)	105 (30)	105 (29)	106 (33)	0.12
HDL, mg/dL	53 (16)	56 (17)	56 (17)	52 (15)	48 (14)	<0.001
LDL, mg/dL	116 (36)	110 (36)	115 (36)	117 (35)	120 (34)	<0.001
Triglyceride, mg/dL	133 (114)	117 (78)	120 (90)	133 (113)	158 (150)	<0.001

Variables	**Overall (47719)** N = 207,536,251	**Q1 (15619)** N = 51,966,212	**Q2 (12606)** N = 51,944,114	**Q3 (10970)** N = 51,881,942	**Q4 (10433)** N = 51,743,982	**p-value**
Total cholesterol, mg/dL	196 (42)	191 (42)	196 (41)	198 (41)	200 (43)	<0.001
Hemoglobin, g/L	143 (15)	126 (12)	139 (8)	147 (7)	159 (9)	<0.001
RDW (%)	13.03 (1.24)	14.27 (1.65)	12.99 (0.68)	12.62 (0.61)	12.25 (0.54)	<0.001
HRR (g/L/%)	11.08 (1.63)	8.96 (1.20)	10.72 (0.30)	11.67 (0.27)	12.99 (0.64)	<0.001

Abbreviation: Q1: Quartile 1; Q2: Quartile 2; Q3: Quartile 3; Q4: Quartile 4; N: weighted number in each group; HDL, High-Density Lipoprotein; LDL, Low-Density Lipoprotein; RDW, Red Cell Distribution Width; HRR, Hemoglobin-to-Red Cell Dis-tribution Width (RDW) ratio; Mean (SD) for continuous variable and weighted number (percentage) for categorical variable.

Supplementary Table S2. Patient characteristics, stratified by all-cause death.

**Variables**	**Overall (47719)** N = 207,540,003	**Survivor (41899)** N = 189,102,339	**Non-Survivor (7730)** N = 18,437,664	**p-value**
**Age, years**	47.2 (17.0)	44.7 (15.6)	66.5 (14.6)	<0.001
**Female**	25,780 (52%)	22,338 (52%)	3,441 (49%)	<0.001
**Race**				<0.001
Non-Hispanic White	22,089 (69%)	17,427 (67%)	4,662 (79%)	
Non-Hispanic Black	10,131 (11%)	8,646 (11%)	1,484 (11%)	
Other Hispanic	4,094 (5.6%)	3,769 (5.9%)	325 (3.4%)	
Mexican American	8,776 (8.2%)	7,772 (8.8%)	1,004 (3.7%)	
Other Races	4,539 (6.9%)	4,284 (7.3%)	255 (3.8%)	
**Education**				<0.001
Below high school	13,438 (17%)	10,355 (16%)	3,082 (31%)	
High school or above	36,121 (83%)	31,503 (84%)	4,618 (69%)	
**Marital status**				<0.001
Married/Living with partner	29,718 (64%)	25,793 (65%)	3,924 (54%)	
Widowed/Divorced/Separated	10,943 (19%)	7,838 (16%)	3,105 (38%)	
Never married	8,488 (17%)	7,940 (19%)	548 (8.0%)	
**Drinking**				<0.001
Never	6,579 (12%)	5,356 (11%)	1,223 (16%)	

Variables	Overall (47719)<br>N = 207,540,003	Survivor (41899)<br>N = 189,102,339	Non-Survivor (7730)<br>N = 18,437,664	p-value
Mild	14,692 (36%)	12,518 (36%)	2,174 (32%)	
Moderate	6,607 (17%)	6,035 (18%)	572 (9.2%)	
Heavy	8,832 (21%)	8,111 (22%)	721 (11%)	
Former	7,951 (15%)	5,523 (12%)	2,427 (32%)	
**Smoking**				<0.001
Never	26,940 (54%)	23,814 (56%)	3,126 (39%)	
Former	12,315 (25%)	9,294 (23%)	3,021 (37%)	
Now	10,331 (21%)	8,761 (21%)	1,569 (23%)	
**Vascular diseases**				
Diabetes	8,665 (13%)	6,189 (11%)	2,476 (28%)	<0.001
Hypertension	21,295 (38%)	15,608 (34%)	5,687 (71%)	<0.001
Hyperlipidemia	35,621 (71%)	29,481 (69%)	6,139 (81%)	<0.001
Chronic kidney diseases	9,133 (15%)	5,515 (11%)	3,618 (44%)	<0.001
**Laboratory tests**				
White blood cells, 1000 cells/ul	7.30 (2.50)	7.27 (2.42)	7.49 (3.04)	<0.001
Platelet count, 1000 cells/ul	254 (66)	254 (64)	248 (79)	<0.001
Glucose, mg/dL	105 (31)	104 (28)	117 (47)	<0.001
HDL, mg/dL	53 (16)	53 (16)	53 (18)	0.006

Variables	Overall (47719)<br>N = 207,540,003	Survivor (41899)<br>N = 189,102,339	Non-Survivor (7730)<br>N = 18,437,664	p-value
LDL, mg/dL	116 (36)	116 (35)	114 (38)	0.018
Triglyceride, mg/dL	133 (114)	130 (115)	154 (103)	<0.001
Total cholesterol, mg/dL	196 (42)	196 (41)	199 (46)	<0.001
Hemoglobin, g/L	143 (15)	143 (15)	141 (16)	<0.001
RDW (%)	13.03 (1.24)	12.99 (1.19)	13.38 (1.53)	<0.001
HRR (g/L/%)	11.08 (1.63)	11.14 (1.60)	10.67 (1.78)	<0.001

Abbreviation: N: weighted number in each group; HDL, High-Density Lipoprotein; LDL, Low-Density Lipoprotein; RDW, Red Cell Distribution Width; HRR, Hemoglobin-to-Red Cell Dis-tribution Width (RDW) ratio; Mean (SD) for continuous variable and weighted number (unweighted percentage) for categorical variable.

Supplementary Table S3. The value of hemoglobin, RDW, and HRR grouped by inflammation status, anemia status, and comorbidities including diabetes, hypertension, hyperlipidemia, and chronic kidney disease.

	Hemoglobin	P	RDW	P	HRR	P
**CRP**		<0.001		<0.001		<0.001
CRP≤0.6mg/dl	144 (15)		12.89 (1. 16)		11.31 (1.59)	
CRP > 0.6mg/dl	139 (15)		13.35 (1.44)		10.52 (1.67)	
**Anemia**		<0.001		<0.001		<0.001
Without anemia	145 (13)		12.84 (0.98)		11.39 (1.38)	
With anemia	113 (11)		14.99 (2.40)		7.74 (1.51)	
**Diabetes**		<0.001		<0.001		<0.001
Without diabetes	144 (14)		12.97 (1.21)		11.18 (1.59)	
With diabetes	141 (16)		13.46 (1.36)		10.59 (1.74)	
**Hypertension**		<0.001		<0.001		<0.001
Without hypertension	144 (14)		12.89 (1.19)		11.25 (1.58)	
With hypertension	142 (15)		13.26 (1.30)		10.87 (1.67)	
**Hyperlipidemia**		<0.001		<0.001		0.200
Without hyperlipidemia	142 (15)		12.98 (1.29)		11.06 (1.64)	
With hyperlipidemia	143 (15)		13.06 (1.22)		11.09 (1.63)	
**Chronic kidney diseases**		<0.001		<0.001		<0.001
Without chronic kidney diseases	144 (14)		12.95 (1.16)		11.20 (1.57)	
With chronic kidney diseases	139 (17)		13.49 (1.51)		10.44 (1.77)	

Abbreviations: RDW: Red Cell Dis-tribution Width ratio; HRR: Hemoglobin-to-Red Cell Dis-tribution Width ratio; CRP: C-reactive Protein.

Supplementary Table S4. Sensitivity analysis after adding CRP or anemia in multivariate analysis of HRR predicting various cardiovascular diseases.

	**Model 1 Adding CRP**<br>**OR (95 % CI)**	**Model 2 Adding anemia**<br>**OR (95 % CI)**
Cardiovascular diseases	0.85 (0.82, 0.88), <0.001	0.83 (0.79, 0.87), <0.001
Stroke	0.95 (0.93, 0.97), <0.001	0.94 (0.94, 0.97), <0.001
Congestive heart failure	0.91 (0.89, 0.93), <0.001	0.90 (0.88, 0.92), <0.001
ASCVD	0.87 (0.84, 0.91), <0.001	0.86 (0.82, 0.90), <0.001
Coronary artery disease	0.94 (0.92, 0.97), <0.001	0.93 (0.91, 0.96), <0.001

Abbreviations: OR, Odds Ratio; 95%CI, 95% Confidence Interval; HDL, High-Density Lipoprotein; LDL, Low-Density Lipoprotein; CRP: C-reactive Protein; ASCVD: Atherosclerotic Cardiovascular Diseases.

Model 1 adjusted for age, sex, race, education, marital status, drinking, smoke, diabetes, hyperten-sion, hyperlipidemia, chronic kidney diseases, white blood cells, platelet count, glucose, HDL, LDL, total cholesterol, triglyceride, CRP.
Model 2 adjusted for age, sex, race, education, marital status, drinking, smoke, diabetes, hyperten-sion, hyperlipidemia, chronic kidney diseases, white blood cells, platelet count, glucose, HDL, LDL, total cholesterol, triglyceride, anemia.

Supplementary Table S5. Sensitivity analysis after adding CRP or anemia in multivariate analysis of HRR predicting various deaths.

	Model 1 Adding CRP<br>HR (95 % CI)	Model 2 Adding anemia<br>HR (95 % CI)
All-cause death	0.83 (0.80, 0.86), <0.001	0.84 (0.80, 0.88), <0.001
Cardiovascular death	0.82 (0.77, 0.87), <0.001	0.83 (0.76, 0.90), <0.001
Non-cardiovascular death	0.84 (0.80, 0.88), <0.001	0.85 (0.80, 0.90), <0.001

Abbreviations: HR,Hazard Ratio; 95%CI, 95% Confidence Interval; HDL, High-Density Lipoprotein; LDL, Low-Density Lipoprotein; CRP: C-reactive Protein; ASCVD: Atherosclerotic Cardiovascular Diseases.

Model 1 adjusted for age, sex, race, education, marital status, drinking, smoke, diabetes, hyperten-sion, hyperlipidemia, chronic kidney diseases, white blood cells, platelet count, glucose, HDL, LDL, total cholesterol, triglyceride, CRP.

Model 2 adjusted for age, sex, race, education, marital status, drinking, smoke, diabetes, hyperten-sion, hyperlipidemia, chronic kidney diseases, white blood cells, platelet count, glucose, HDL, LDL, total cholesterol, triglyceride, anemia.

Supplementary Table S6. Subgroup analysis grouped by age, sex, race, inflammation status, anemia status, and comorbidities including diabetes, hypertension, hyperlipidemia, and chronic kidney disease.

	OR (95%CI), P-value	p for interaction	HR (95%CI), P-value	p for interaction
	**Cardiovascular disease**		**All-cause death**	
**Age**		0.270		0.100
The young	0.82(0.77,0.86), <0.001		0.84(0.79,0.88), <0.001	
The old	0.87(0.82,0.92), <0.001		0.81(0.78,0.84), <0.001	
**Sex**		**0.040**		**<0.001**
Male	0.73(0.69,0.78), <0.001		0.66(0.63,0.68), <0.001	
Female	0.80(0.76,0.85), <0.001		0.85(0.81,0.89), <0.001	
**Race**		0.350		0.140
Non-Hispanic White	0.78(0.74,0.83), <0.001		0.76(0.73,0.79), <0.001	
Non-Hispanic Black	0.82(0.78,0.87), <0.001		0.80(0.75,0.85), <0.001	
Other Hispanic	0.85(0.75,0.97), 0.020		0.91(0.75,1.10), 0.32	
Mexican American	0.82(0.73,0.92), <0.001		0.80(0.74,0.87), <0.001	
Other Races	0.83(0.64, 1.08), 0.160		0.75(0.62, 0.90), 0.002	
**CRP**		0.300		0.900
CRP≤0.6mg/dl	0.86(0.80,0.92), <0.001		0.83(0.80,0.87), <0.001	
CRP > 0.6mg/dl	0.87(0.79,0.95), 0.002		0.82(0.77,0.89), <0.001	
**Anemia**		0.730		0.340
Without anemia	0.85(0.78,0.91), <0.001		0.85(0.81,0.89), <0.001	
With anemia	0.94(0.83,1.06), 0.320		0.76(0.69,0.84), <0.001	
**Diabetes**		0.450		0.720
Without diabetes	0.80(0.76,0.84), <0.001		0.79(0.76,0.82), <0.001	
With diabetes	0.84(0.78,0.90), <0.001		0.79(0.75,0.84), <0.001	
**Hypertension**		0.100		0.040
Without hypertension	0.87(0.81,0.93), <0.001		0.83(0.79,0.88), <0.001	
With hypertension	0.82(0.78,0.86), <0.001		0.80(0.77,0.83), <0.001	

**Hyperlipidemia**		0.510		0.320
Without hyperlipidemia	0.80(0.73,0.88), <0.001		0.78(0.74,0.83), <0.001	
With hyperlipidemia	0.82(0.78,0.85), <0.001		0.80(0.78,0.83), <0.001	
**Chronic kidney diseases**		**0.020**		0.200
Without chronic kidney diseases	0.87(0.83,0.91), <0.001		0.85(0.81,0.89), <0.001	
With chronic kidney diseases	0.79(0.74,0.85), <0.001		0.84(0.80,0.88), <0.001	

Abbreviation: OR, Odds Ratio; HR,Hazard Ratio; 95%CI, 95% Confidence Interval; CRP: C-reactive Protein

Model adjusted for age, sex, race, education, marital status, drinking, smoke, diabetes, hyperten-sion, hyperlipidemia, chronic kidney diseases, white blood cells, platelet count, glucose, HDL, LDL, total cholesterol, triglyceride. For each subgroup analyses, the stratification factors used to identify subgroups are not adjusted for in the multivariate regression model.

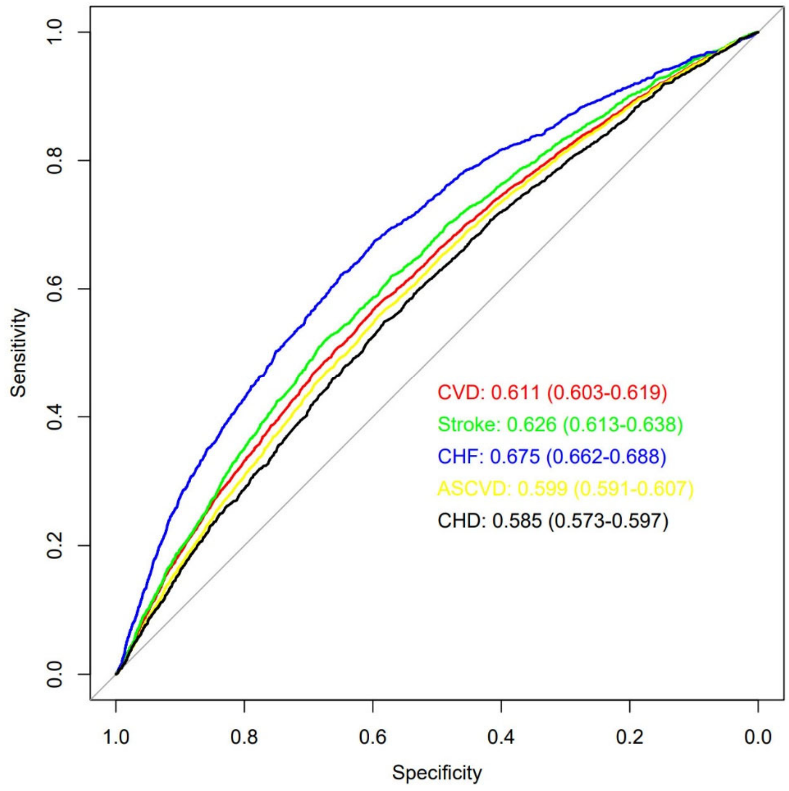

Supplementary Figure S1. The Receiver Operating Characteristic (ROC) curve of HRR (Hemoglobin-to-Red Cell Dis-tribution Width ratio) for predicting cardiovascular diseases.

Abbreviation: CVD, Cardiovascular Diseases; CHF, Congestive Heart Failure; ASCVD: Atherosclerotic Cardiovascular Diseases; CHD: Coronary Heart Disease.

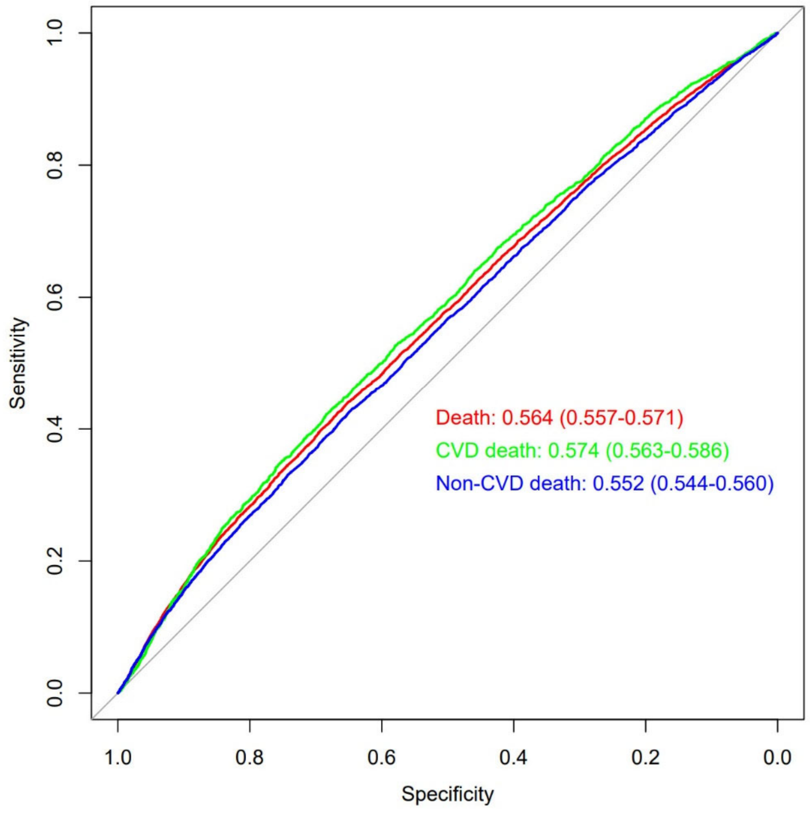

Supplementary Figure S2. The Receiver Operating Characteristic (ROC) of HRR (Hemoglobin-to-Red Cell Dis-tribution Width ratio) for predicting death.

Abbreviation: CVD death: Cardiovascular Death; Non-CVD death: Non-cardiovascular Death.
